# Influence of diagnosis of gestational diabetes mellitus on fear of childbirth

**DOI:** 10.1016/j.clinsp.2024.100501

**Published:** 2024-09-14

**Authors:** Cibele Santini de Oliveira Imakawa, Silvana Maria Quintana, Geraldo Duarte, Elaine Christine Dantas Moisés

**Affiliations:** a Department of Gynecology and Obstetrics, Faculdade de Medicina de Ribeirão Preto, Universidade de São Paulo, Ribeirão Preto, SP, Brazil; b Ribeirão Preto Women's Health Reference Center, Ribeirão Preto, SP, Brazil

**Keywords:** High-risk pregnancy, Gestational diabetes mellitus, Fear of childbirth, Fear of birth scale, Parturition

## Abstract

**Introduction:**

Considering the relationship between Gestational Diabetes Mellitus and maternal and perinatal adverse outcomes, it's pertinent to investigate whether this diagnosis is a predictor of fear of childbirth. As there is little data about the fear of childbirth in Brazil, it´s necessary to understand better the population, and this way the authors can identify factors that influence this fear as well as propose public health policies to treat it.

**Objective:**

The main goal was to compare the prevalence of fear of childbirth between the groups of low-risk pregnancy and gestational diabetes mellitus.

**Material and methods:**

In this cohort study, the sample consisted of 319 patients divided into low-risk pregnancy group (n = 152) and gestational diabetes mellitus group (n = 167). Patients have undergone a semi-structured interview with epidemiological, obstetric, and anthropometric data and the main cause of fear of childbirth. In addition, the patients have marked an “X” on the scale into the Fear of Birth Scale to describe their fear. After delivery, data have been collected through electronic medical records.

**Results:**

The prevalence of fear of childbirth found was higher for the gestational diabetes mellitus group (46.05%) compared to the low-risk pregnancy group (34.73%) with a cutoff score ≥ 54. In the whole sample, the main cause of fear of childbirth (score ≥ 60) was the pain of labor and delivery (31.58%).

**Conclusions:**

The prevalence of fear of childbirth in the present study was greater than 30%, highlighting the relevance of implementing this assessment during prenatal care.

## Highlights

• Fear of childbirth is more prevalent in patients with gestational diabetes mellitus.
• Fear of Birth Scale score is higher in patients with gestational diabetes mellitus.
• Reasons related to vaginal birth are more associated with clinically relevant fear.

## Introduction

The definitions of Fear of Childbirth (FOC), worry, and anxiety are controversial, and no standardized tools to assess FOC have been reported.^1^ Up to 80% of pregnant women experience concerns and fears.^2^^,^^3^ Tokophobia, a severe fear of childbirth, affects 7%–25% of primiparous women, 7.7%–16.25% of multiparous women, and 7%–18.6% of women with tokophobia desire an elective cesarean section.^4^ FOC is associated with increased anxiety and depression during pregnancy, an increased risk of premature labor, increased labor duration, and an increased risk of developing depression and post-traumatic stress disorder after childbirth. Several factors that protect against or aggravate FOC have been identified, though some factors remain controversial.^5^

However, the effect of the diagnosis of high-risk pregnancy on FOC has not yet been reported. Diabetes mellitus is characterized by persistent hyperglycemia caused by deficient insulin production, inadequate insulin release, and/or peripheral resistance to insulin.^6^, ^7^, ^8^ Hyperglycemia detected during pregnancy can be divided into GDM and diabetes in pregnancy, which can be classified as pre-gestational diabetes or diabetes diagnosed for the first time during pregnancy when it meets the criteria for non-gestational diabetes.^9^, ^10^, ^11^

Considering the high worldwide prevalence of GDM, affecting an average of 16.7% of pregnant women and being one of the main clinical conditions during the pregnancy-puerperal cycle, it is essential to assess whether the gestational risk resulting from the development of GDM influences fear of childbirth.^12^ This way, the main goal of this study was to evaluate the influence of the diagnosis of gestational diabetes mellitus on fear of childbirth.

## Participants, ethics and methods

This cohort study was approved by the Research Ethics Committee (REC) of the Clinics Hospital of the School of Medicine of Ribeirão Preto, University of São Paulo (*Hospital das Clínicas da Faculdade de Medicina da Universidade de São Paulo*; HCFMRP-USP) and did not interfere with the obstetric management of the women (approval n° 3,712,476). The present study adhered to the STROBE guidelines (Strengthening the Reporting of Observational Studies in Epidemiology).^13^

Women aged 18 years or older with a single pregnancy at gestational age ≥ 34 weeks who were undergoing prenatal follow-up care at the Reference Center for Women's Health of Ribeirão Preto or at the HCFMRP-USP from January 2020 to October 2022 were included in this study. The women were divided into the Low-Risk Pregnancy Group (LRP) and the GDM group.

The diagnosis of GDM was established when one or more blood glucose values were altered in the oral glucose tolerance test, according to the following parameters: fasting blood glucose, one and two hours after an overload of 75g of anhydrous glucose, respectively, greater than or equal to 92 and less than or equal to 125 mg/dL, greater than or equal to 180 mg/dL, greater than or equal to 153 and less than or equal to 199 mg/dL.^14^^,^^15^ The LRP was the control group and consisted of women without maternal comorbidities and without fetal and/or placental attachment changes.

All women provided informed consent and were literate. The interviews were conducted during the women's third-trimester prenatal follow-up visits at the research centers. In-person interviews based on a semi-structured questionnaire regarding the epidemiological, obstetric, and anthropometric data of the women were conducted. Subsequently, the women answered the instruments of the Brazilian Economic Classification Criteria, the Fear of Birth Scale (FOBS), and the specific question about the main cause of FOC.

**Fig. 1:**
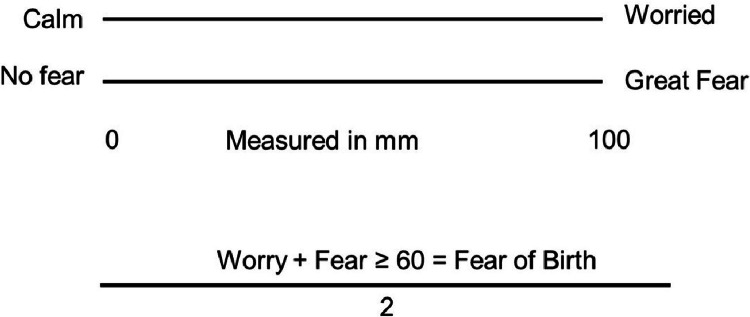
Fear of childbirth scale.^19^, ^20^, ^21^

The FOBS included the item, “How do you feel about the upcoming childbirth now?” The women were instructed to place a mark on two 100 mm Visual Analog Scales (VASs) that have the anchor words calm/concern and no fear/great fear. The FOBS score is calculated as the mean score of those two scales about concern and fear^16^ (Fig. 1). According to previous studies, the cutoff of mean ≥ 54 mm and ≥ 60 mm was considered clinically significant.^1^^,^^16^, ^17^, ^18^

After childbirth, the women's obstetric data were retrospectively obtained from electronic medical records. The data were collected from January 2020 to November 2022. Intending to guarantee the confidentiality of the information, the main researcher was the only one who had access to the database with the identification of the women. After obtaining the obstetric data, the numbers of the women's medical records were replaced by a code containing the classification between the control and study groups, in addition to the sequential numbering of inclusion, which was maintained in the definitive database.

This is the first study regarding the risk of FOC in women with GDM. Thus, the necessary sample size was calculated according to the risk of FOC in women with uncomplicated pregnancies (80%).^2^^,^^3^ Assuming a 15% increase in fear of childbirth-related to GDM as a clinically significant difference and a test power of 80%, the necessary sample size was calculated using SAS software (version 9.4, SAS Institute, North Carolina) using the proc power procedure. In this cohort study, a total of 304 women are necessary, including 152 in each group. As some women were predicted to be lost due to giving birth at another institution, a safety margin of 10% was added for the phase two analysis, increasing each group to 167 women to allow for an analysis of secondary variables.

Boxplot and histogram graphs were performed to verify the distribution of quantitative variables in relation to groups (LRP and GDM). Qualitative variables were summarized considering absolute and relative frequencies.

To verify if there is a statistical relevance of the quantitative variables in relation to the study groups, the Wilcoxon test for independent samples was applied. This test was chosen due to data distribution.

The Chi-Square test was applied to verify if there is an association between the qualitative variables in relation to the study groups.

The reliability of the Fear of Birth Scale was estimated considering Cronbach's alpha coefficient. This coefficient ranges from zero to one, with measures close to one indicating greater reliability of the scale.

Pearson's coefficient was calculated to verify the correlation between worry and fear.

Statistical analyses were implemented in the SAS version 9.4 program.

## Results

**Fig. 2:**
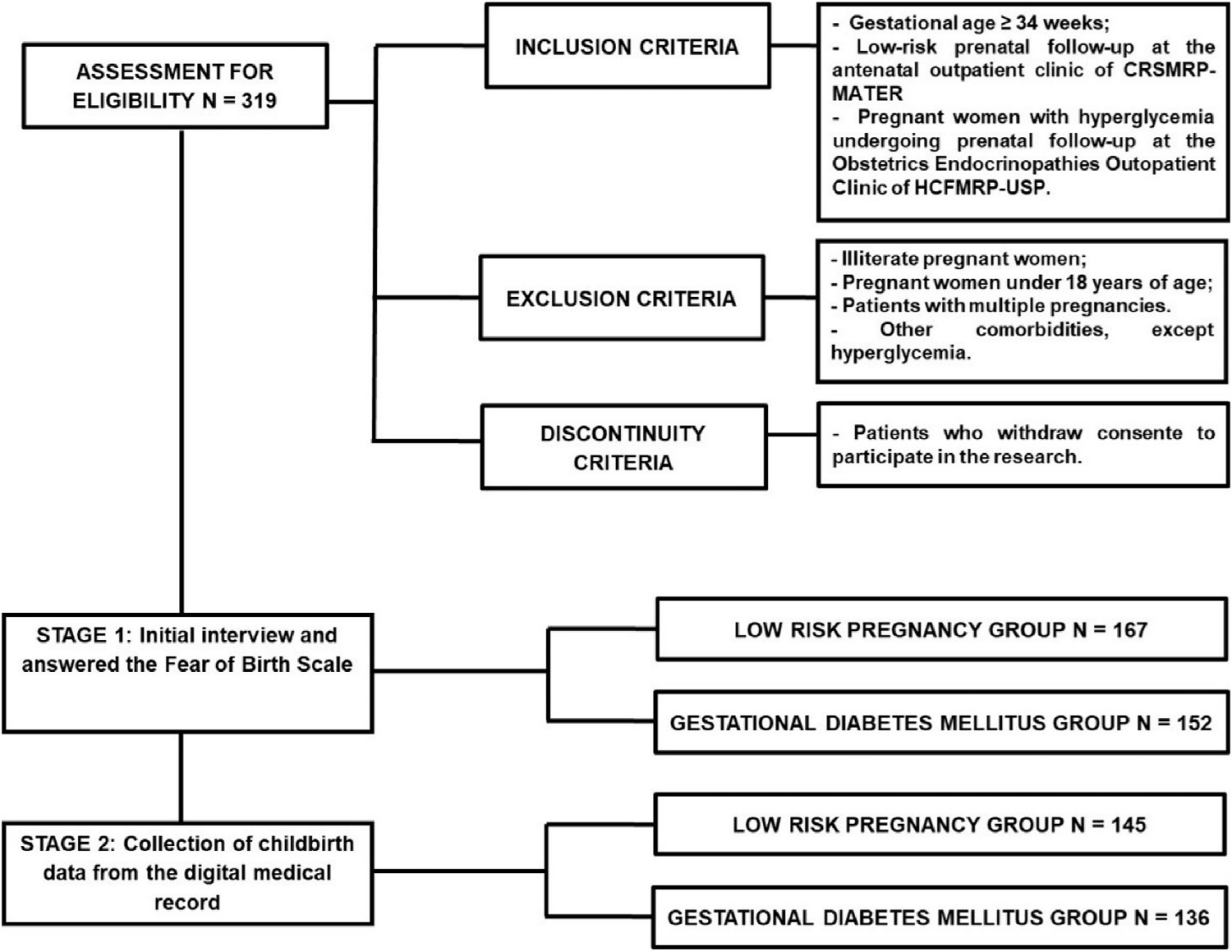
Woman flowchart.

A total of 319 women underwent a semi-structured interview and completed the FOBS, including 167 women in the LRP group and 152 in the GDM group (Fig. 2).

The mean women's age was 25.69 ± 5.33 years in the LRP group and 28.92 ± 6.25 years in the GDM group (p = 0.0001). The mean gestational age at the time of the interview was higher in the LRP group (36.83 ± 1.25 weeks) than in the GDM group (36.47 ± 1.46 weeks) (p = 0.0402) (Table 1). women in the GDM group had significantly more pregnancies (2.47 ± 1.41 pregnancies) than those in the LRP group (1.97 ± 1.11 pregnancies) (p = 0.0008). The mean parity was 1.26 ± 1.33 in the GDM group and 0.80 ± 0.96 in the LRP group (p = 0.0007). The number of abortions was not significantly different between the groups. While the woman's height was not significantly different between the groups, the pre-gestational weight (p = 0.0001), pre-gestational Body Mass Index (BMI) (p = 0.0001), gestational weight (p = 0.0004), and gestational BMI (p = 0.0003) were higher in the GDM group.

**Table 1:** Women characteristics.

		LRP (n = 167)	GDM (n = 152)	p-value
**Obstetric history and parity**	Maternal age (years)	25.69 (± 5.33)	28.92 (± 6.25)	0.0001
	GA (weeks)	36.83 (± 1.25)	36.47 (± 1.46)	0.0402
	Number of pregnancies	1.97 (± 1.11)	2.47 (± 1.41)	0.0008
	Parity	0.80 (± 0.96)	1.26 (± 1.33)	0.0007
	Number of abortions	0.17 (± 0.44)	0.20 (± 0.47)	0.5710
**Anthropometric data**	Height (meters)	1.62 (± 0.07)	1.62 (± 0.06)	0.7630
	Pre-gestational weight (kg)	65.46 (± 13.84)	73.94 (± 17.42)	0.0001
	Pre-gestational BMI (kg/m^2^)	24.86 (± 4.71)	28.09 (± 6.09)	0.0001
	Gestational weight (kg)	77.70 (± 13.85)	83.81 (± 16.18)	0.0004
	Gestational BMI (kg/m^2^)	29.52 (± 4.58)	31.85 (± 5.62)	0.0003

Data are presented as mean ± standard deviation.

GDM, Gestational Diabetes Mellitus; LRP, Low Risk Pregnancy; GA, Gestational Age; kg, Kilograms; BMI, Body Mass Index; m^2^, Square meter.

*p-value referring to the Wilcoxon test for independent samples.

The demographic and socioeconomic variables were similar between the groups (Table 2). The history of fetal death was less than 5% in both groups and the majority of women reported not planning their current pregnancy.

**Table 2:** Qualitative women characteristics.

		LRP (n = 167)	GDM (n = 152)	p-value
**Race**	Non-white	65 (38.92%)	59 (38.82%)	0.9845
	White	102 (61.08%)	93 (61.18%)	
**Profession**	Unpaid	92 (55.09%)	88 (57.89%)	0.6138
	Paid	75 (44.91%)	64 (42.11%)	
**MS**	Without partner	27 (16.17%)	14 (9.21%)	0.0637
	With partner	140 (83.83%)	138 (90.79%)	
**Years of study**	< 8 years	20 (11.98%)	23 (15.13%)	0.2153
	8 years	11 (6.59%)	6 (3.95%)	
	> 8 and < 11 years	42 (25.15%)	40 (26.32%)	
	11 years	70 (41.92%)	50 (32.89%)	
	> 12 years	24 (14.37%)	33 (21.71%)	
**PFD**	No	162 (97.01%)	151 (99.34%)	0.1250
	Yes	5 (2.99%)	1 (0.66%)	
**PP**	No	107 (64.07%)	89 (58.55%)	0.3118
	Yes	60 (35.93%)	63(41.45%)	
**PH**	No	155 (92.81%)	135 (88.82%)	0.2147
	Yes	12 (7.19%)	17 (11.18%)	
**PF**	No	11 (91.67%)	15 (88.24%)	0.7651
	Yes	1 (8.33%)	2 (11.76%)	

GDM, Gestational Diabetes Mellitus; LRP, Low Risk Pregnancy; MS, Marital Status; PFD, Previous Fetal Death; PP, Planned Pregnancy; PH, Psychiatric History; PF, Psychiatric Follow-up.

*p-value referring to the Chi-Square test.

The most prevalent economic class in the LRP group was C2, in which the monthly household income is USD 330.55, comprising 35.93% of the group. The most prevalent economic class in the GDM group was C1, in which the monthly household income is USD 583.27, comprising 35.53% of the group (Table 3).

**Table 3:** Socioeconomic classification.

	Household income (USD)	LRP (n = 167)	GDM (n = 152)	p-value
**A**	4.830.69	1(0.60%)	2 (1.32%)	0.8790
**B1**	2.132.16	4 (2.40%)	2 (1.32%)	
**B2**	1.066.47	25 (14.97%)	21 (13.82%)	
**C1**	583.27	54 (32.34%)	54 (35.53%)	
**C2**	330.55	60 (35.93%)	49 (32.24%)	
**D‒E**	136.07	23 (13.77%)	24 (15.79%)	

Modified ABEP (*Associação Brasileira de Empresas de Pesquisa* ‒ Brazilian Association of Market Research Companies).

GDM, Gestational Diabetes Mellitus; LRP, Low Risk Pregnancy.

*p-value referring to the Chi-Square test.

The worry and total FOBS scores were higher in the GDM group while the fear score was not significantly different between the groups (Table 4).

**Table 4:** FOBS scores.

	LRP (n = 167)			GDM (n = 152)			p-value
	Mean ± SD	Median (interquartile range)	Range	Mean ± SD	Median (interquartile range)	Range	
**Worry**	41.57±33.28	41.5 (8.5‒69.0)	0-100	49.38±33.27	50 (17.5‒78.75)	0‒100	0.0440
**Fear**	48.13±33.53	47 (16–80)	0.5‒100	53.23±31.20	54 (28.5‒82)	1‒100	0.2094
**Total score**	44.85±31.30	46.63 (17.75–68.75)	0.75‒100	51.30±28.90	51.13 (28.25‒75.38)	1.25‒100	0.0489

GDM, Gestational Diabetes Mellitus; LRP, Low Risk Pregnancy; SD, Standard Deviation.

*p-value referring to the Wilcoxon test for independent samples.

More women in the GDM group met the cutoff value of 54 for the worry VAS (45.39%), fear VAS (50%), and total score VAS (46.05%) than in the LRP group (32.34%, 38.92%, and 34.73%, respectively; p = 0.0167, p = 0.0466, and p = 0.0393, respectively). More women in the GDM group met the cutoff value of 60 for worry VAS (GDM = 41.25%, LRP = 29.34%, p = 0.0237) (Table 5).

**Table 5:** Clinically significant concern and fear regarding fear of childbirth.

Fear of childbirth Scale	Cutoff point		LRP (n = 167)	GDM (n = 152)	p-value
Worry	≥ 54	No	113 (67.66%)	83 (54.61%)	0.0167
		Yes	54 (32.34%)	69 (45.39%)	
	≥ 60	No	118 (70.66%)	89 (58.55%)	0.0237
		Yes	49 (29.34%)	63 (41.45%)	
Fear	≥ 54	No	102 (61.08%)	76 (50%)	0.0466
		Yes	65 (38.92%)	76 (50%)	
	≥ 60	No	110 (65.87%)	87 (57.24%)	0.1131
		Yes	57 (34.13%)	65 (42.76%)	
Total score	≥ 54	No	109 (65.27%)	82 (53.95%)	0.0393
		Yes	58 (34.73%)	70 (46.05%)	
	≥ 60	No	114 (68.26%)	91 (59.87%)	0.1181
		Yes	53 (31.74%)	61 (40.13%)	

GDM, Gestational Diabetes Mellitus; LRP, Low Risk Pregnancy.

*p-value referring to the Chi-Square test.

The relative risk of FOC (measured using a cutoff value of 54) in women with GDM is 1.33 (95% Confidence Interval: 1.0124–1.7368). The relative risk of FOC (measured using a cutoff value of 60) in women is 1.26 (95% CI: 0.9412–1.6989).

The two main reasons for FOC in the groups were fear regarding fetal death and suffering and fear regarding labor and childbirth (Table 6).

**Table 6:** Reasons for FOC.

	LRP (n = 167)	GDM (n = 152)	p-value
**Fetal death/suffering**	48 (28.74%)	52 (34.21%)	0.3737
**Pain of labor and childbirth**	39 (23.35%)	40 (26.32%)	
**Not being able to give birth**	14 (8.38%)	17 (11.18%)	
**Not being able to deal with the lack of control**	11 (6.59%)	6 (3.95%)	
**Unable to raise the child**	3 (1.80%)	2 (1.32%)	
**Lack of trust in the team**	2 (1.20%)	3 (1.97%)	
**Going through an unwanted procedure or situation**	28 (16.77%)	18(11.84%)	
**Having another bad experience**	9 (5.39%)	10 (6.58%)	
**No fear of childbirth**	13 (7.78%)	4 (2.63%)	

FOC, Childbirth Fear; GDM, Gestational Diabetes Mellitus; LRP, Low Risk Pregnancy.

*p-value referring to the Chi-Square test.

Clinically significant FOC (measured using a cutoff value of 60) was associated with the white race (Table 7, Table 8). There were no differences in obstetric outcomes between women with and without FOC (Table 9).

**Table 7:** Characteristics of women with and without clinically significant fear of childbirth.

	< 60 (n = 205)			≥ 60 (n = 114)			p-value
	Mean ± SD	Median (interquartile range)	Range	Mean ± SD	Median (interquartile range)	Range	
**Age (years)**	26.96 ± 6.04	26 (22‒30)	18‒43	27.72 ± 5.92	28 (23‒32)	18‒41	0.1852
**GA (weeks)**	36.77 ± 1.40	37 (36‒38)	34‒41	36.46 ± 1.29	36 (36‒37)	34‒40	0.0480
**Number of pregnancies**	2.14 ± 1.25	2 (1 ‒3)	1‒7	2.32 ± 1.33	2 (1‒3)	1‒8	0.2110
**Childbirths**	0.98 ± 1.15	1 (0 ‒1)	0‒6	1.09 ± 1.22	1 (0‒2)	0‒7	0.4211
**Number of Abortions**	0.16 ± 0.39	0 (0)	0‒2	0.24 ± 0.54	0 (0)	0‒3	0.2999
**Pre-gestational weight (kg)**	68.35 ± 15.12	66 (58‒76)	32.60‒130	71.56 ± 17.83	69.5 (60‒80)	39‒144	0.1925
**Height (m)**	1.62 ± 0.06	1.62 (1.57 ‒1.65)	1.40‒1.82	1.63 ± 0.07	1.62 (1.57‒1.68)	1.48‒1.81	0.3485
**Pre-gestational BMI (Kg/m^2^)**	26.09 ± 5.33	25.10 (22.64–29.05)	13.39‒48.33	26.96 ± 6.12	26.49 (22.77‒30.43)	15.05‒47.02	0.2098
**Gestational weight**	79.66 ± 14.54	79 (70 – 88)	42.40‒143	82.31 ± 16.48	79 (70 – 90)	45‒136.80	0.2274
**Gestational BMI (kg/m^2^)**	30.43 ± 5.04	29.97 (26.45‒33.53)	18.84‒53.17	30.99 ± 5.55	30.94 (27.24 – 34.81)	17.36‒47.90	0.3224

SD, Standard Deviation; GA, Gestational Age; BMI, Body Mass Index.

*p-value referring to the Wilcoxon test for independent samples.

**Table 8:** Qualitative characteristics of women with and without clinically significant childbirth fear.

		< 60 (n = 205)	≥ 60 (n = 114)	p-value
**Race**	Non-white	70 (34.15%)	54 (47.37%)	0.0203
	White	135 (65.85%)	60 (52.63%)	
**Maternal Age**	19 years	17 (8.29%)	11 (9.65%)	0.9071
	≥ 19 e < 35	163 (79.51%)	90 (78.95%)	
	≥ 35	25 (12.20%)	13 (11.40%)	
**BMI**	< 25	98 (47.80%)	44 (38.60%)	0.1128
	≥ 25	107 (52.20%)	70 (61.40%)	
**Abortion**	No	174 (84.88%)	92 (80.70%	0.3369
	Yes	31 (15.12%	22 (19.30%)	
**Profession**	Unpaid	115 (56.10%)	65 (57.02%)	0.8738
	Paid	90 (43.90%)	49 (42.98%)	
**Marital status**	Without partner	27 (13.17%)	14 (12.28%)	0.8199
	With partner	178 (86.83%)	100 (87.72%)	
**Years of study**	< 8 years	27 (13.17%)	16 (14.04%)	0.8089
	8 years	11 (5.37%)	6 (5.26%)	
	> 8 and < 11 years	57 (27.80%)	25 (21.93%)	
	11 years	76 (37.07%)	44 (38.60%)	
	> 12 years	34 (16.59%)	23 (20.18%)	
**PFD**	No	201 (98.05%)	112 (98.25%)	0.9013
	Yes	4 (1.95%)	2 (1.75%)	
**PP**	No	125 (60.98%)	71 (62.28%)	0.8185
	Yes	80 (39.02%)	43 (37.72%)	
**PH**	No	191 (93.17%)	99 (86.84%)	0.0595
	Yes	14 (6.83%)	15 (13.16%)	
**PF**	No	13 (92.86%)	13 (86.67%)	0.5844
	Yes	1 (7.14%)	2 (13.33%)	
**Socioeconomic classification (USD)**	4.830.69	2 (0.98%)	1 (0.88%)	0.9964
	2.132.16	4 (1.95%)	2 (1.75%)	
	1.066.47	31 (15.12%)	15 (13.16%)	
	583.27	69 (33.66%)	39 (34.21%)	
	330.55	70 (34.15%)	39 (34.21%)	
	136.07	29 (14.15%)	18 (15.79%)	

BMI, Body Mass Index before pregnancy; PFD, Previous Fetal Death; PP, Planned Pregnancy; PH, Psychological History of depression; PF, Psychiatric Follow-up for women with a history of depression

*p-value referring to the Chi-Square test.

**Table 9:** Obstetric characteristics of women with and without clinically significant childbirth fear.

Variable		< 60 (n = 179)	≥ 60 (n = 102)	p-value
Childbirth mode	Vaginal	121 (67.60%)	64 (62.75%)	0.4095
	Cesarean section	58 (32.40%)	38 (37.25%)	
Start of labor	Spontaneous	88 (49.16%)	551 (50.49%)	0.9961
	Induced	71 (39.66)	39 (38.61%)	
	Cesarean section without induction	20 (11.17)	11 (10.89%)	
Indication for Cesarean section	No	120 (67.04%)	68 (66.67%)	0.9491
	Yes	59 (32.96%)	34 (33.33%)	
CSR	No	174 (97.20%)	97 (95.10%)	0.3600
	Yes	5 (2.80%)	5 (4.90%)	
Analgesia	No	127 (70.95%)	64 (62.75%)	0.1765
	Yes	52 (29.05%)	38(37.25%)	

CSR, Cesarean Section Request.

*p-value referring to the Chi-Square test.

The internal consistency of the FOC scale was measured by α-Cronbach's Coefficient was good (α-Cronbach's Coefficient = 0.8164). The scales used to measure concern and FOC were correlated (Pearson's correlation coefficient = 0.69).

## Discussion

This is the first study to report the association between the gestational risk of GDM and FOC. The International Federation of Gynecology and Obstetrics estimates that one in six pregnant women has some type of hyperglycemia during pregnancy, including 84% of those with GDM.^22^

The sample consisted of women with higher pre-gestational and gestational weight and BMI; maternal age and parity in the study group, characteristics classically considered risk factors for the development of GDM.^23^

There is no widely used cutoff value of the FOCQ to represent clinically significant FOC. An Australian study used a cutoff value of 54,^20^ while other studies used a cutoff value of 60.^17^^,^^18^ Rondung et al. also used a cutoff value of 60 in a Swedish study and reported a prevalence of clinically significant FOC of 24.6%.^1^ As no single cutoff value has been accepted, both previously reported cutoff values were used in this study. A previous cross-cultural study used the FOCQ to compare the prevalence of FOC among pregnant women living in Sweden and Australia.^16^ While the prevalences of FOC were similar between the groups, a cutoff value of 50 was used in the previous study based on the results of a Finnish study that used a single-item VAS that was the starting point to establish the standard values of the FOCQ.^24^ Other previous studies reported an FOC prevalence of 22% when a cutoff value of 60 was used.^17^ The prevalence of FOC found in this study was higher than previously reported values, regardless of the cutoff value used.

The causes of FOC can be classified as relating to the infant's well-being; procedures and complications throughout the childbirth process; personal issues including insecurity and fear of losing control; and external issues related to the healthcare team.^5^^,^^25^^,^^26^ In this study, the main causes of FOC were fears of fetal death/suffering, labor pain, and experiencing unwanted procedures. Among women with clinically significant FOC, the main causes of concern were labor and childbirth pain (31.58%), fetal death/suffering (26.32%), and not being able to give birth (14.91%). Nearly half (46.49%) of the women with clinically significant FOC in this study had fears related to a vaginal birth.

The association between FOC and elective cesarean section procedures has been studied. In 2022, a systematic review reported that the prevalence of tokophobia ranged from 7%–25% in primiparous women and from 7.7%–16.25% in multiparous women and that approximately 7%–18.6% of women with tokophobia requested elective cesarean sections.^4^. The World Health Organization recommends cesarean section rates between 10%–15%.^27^ However, the cesarean section rate in Brazil is 55% among women of the Unified Health System and 90% among the private sector. A cesarean section is the preferred route of childbirth in 27% of women in the public sector and 44% in the private sector in Brazil.^28^. The maternal preference for cesarean childbirth in Brazil is based on maternal convenience and fear of labor pain,^28^ which are similar to the factors affecting maternal preference in other countries.^29^^,^^30^ Tokophobia is the primary cause of cesarean section requests.^26^^,^^31^ Women who report high FOC are more likely to request a cesarean section.^24^^,^^32^^,^^33^ FOC was associated with a preference for a cesarean section in a previous study.^34^ When this fear is not treated in a timely manner, the chance of a cesarean section is increased five-fold,^35^ resulting in cesarean sections without medical indications and exposing the women to unnecessary risks.^24^^,^^35^ In this study, FOC did not affect the obstetric outcomes, which may be due to the fact that this study was conducted at an accredited public institution that follows the principles of *Hospital Amigo da Criança e da Mulher*.

It is also important to highlight that even though it is a public university and reference hospital in the state of São Paulo, cesarean section rates are around 20% above that recommended by the WHO and therefore it is essential to begin debating and implementing public health policies to identify and assess women's fear of childbirth, as this way they will be less afraid of vaginal birth and it would be possible to reduce cesarean section rates.

## Conclusion

This is the first study to determine the prevalence of FOC by using the Fear of Birth Scale in Brazil and the effects of gestational risk factors, such as GDM, on FOC. The prevalence of FOC is higher in Brazil than in other countries. A diagnosis of GDM is associated with an increased prevalence of FOC. The main causes of FOC include the fear of labor pain and childbirth, the fear of fetal death/suffering, and the fear of not being able to give birth. Therefore, public policies to educate the women about risks and benefits of the route of birth and to assess and treat FOC should be discussed and created.

## Authors’ contributions

Cibele Santini de Oliveira Imakawa: Conceptualization; methodology; validation; formal analysis; investigation; data curation; writing-original draft, writing-review & editing, visualization.

Silvana Maria Quintana: Resources; writing-review & editing; funding acquisition. geraldo duarte: resources; writing-review & editing; funding acquisition.

Elaine Christine Dantas Moisés: Conceptualization; methodology; validation; formal analysis; resources; writing-original draft, writing-review & editing, visualization; supervision; project administration; funding acquisition.

## Declaration of competing interests

The authors declare that they have no known competing financial interests or personal relationships that could have appeared to influence the work reported in this paper.
